# Community Change within a Caribbean Coral Reef Marine Protected Area following Two Decades of Local Management

**DOI:** 10.1371/journal.pone.0054069

**Published:** 2013-01-14

**Authors:** Mae M. Noble, Gregoor van Laake, Michael L. Berumen, Christopher J. Fulton

**Affiliations:** 1 ARC Centre of Excellence for Coral Reef Studies, Research School of Biology, The Australian National University, Canberra, Australia; 2 The Saba Conservation Foundation, The Bottom, Saba, Netherland Antilles; 3 Red Sea Research Center, King Abdullah University of Science and Technology, Thuwal, Kingdom of Saudi Arabia; 4 Biology Department, Woods Hole Oceanographic Institution, Woods Hole, Massachusetts, United States of America; University of Canterbury, New Zealand

## Abstract

Structural change in both the habitat and reef-associated fish assemblages within spatially managed coral reefs can provide key insights into the benefits and limitations of Marine Protected Areas (MPAs). While MPA zoning effects on particular target species are well reported, we are yet to fully resolve the various affects of spatial management on the structure of coral reef communities over decadal time scales. Here, we document mixed affects of MPA zoning on fish density, biomass and species richness over the 21 years since establishment of the Saba Marine Park (SMP). Although we found significantly greater biomass and species richness of reef-associated fishes within shallow habitats (5 meters depth) closed to fishing, this did not hold for deeper (15 m) habitats, and there was a widespread decline (38% decrease) in live hard coral cover and a 68% loss of carnivorous reef fishes across all zones of the SMP from the 1990s to 2008. Given the importance of live coral for the maintenance and replenishment of reef fishes, and the likely role of chronic disturbance in driving coral decline across the region, we explore how local spatial management can help protect coral reef ecosystems within the context of large-scale environmental pressures and disturbances outside the purview of local MPA management.

## Introduction

Marine Protected Areas (MPAs) are often used as a spatial management tool to balance harvesting pressures against the need to conserve biodiversity and maintain key ecosystem processes [1], [2], [3]. One of the many challenges for MPA design and assessment is recognising both the expected benefits and limitations that spatial management can provide [4]. Alterations in size and location, socioeconomic incentives and compliance mechanisms, time since establishment, and the ecological setting can all influence MPA effectiveness [1], [2], [5]–[10]. Decades of MPA implementation have enabled studies of the long-term effects of MPA-based management on reef ecosystems [11]–[13]. While still rare, these decadal-scale examinations have revealed some of the marked benefits of well-managed no-take MPAs in maintaining high biodiversity coral reef ecosystems across the Indo-Pacific [7], [12]–[14]. Studies that have explored how key functional elements of the coral reef community have changed over time have been particularly revealing for our understanding of why changes have occurred across fished and no-take zones [1], [4], [13].

Effects of spatial management on coral reef ecosystems can often take considerable time to be fully realised. For species targeted by fishing, up to six-fold increases in fish density and biomass have been documented within no-take areas, but in many cases this has taken 10 or more years to occur [5], [7], [11]–[13]. Likewise, spillover of both adult fish and new recruits from no-take to fished areas can take considerable time, depending on species-specific demographics and the carrying capacity of habitats [1], [15]–[17]. Extrinsic factors can also drive cyclical changes in fish abundance over decadal time periods [12], often due to periodic disturbances such as hurricanes that can significantly alter coral reef community structure [4], [18]–[21]. While the optimum age of a no-take MPA can depend on many factors, modelled estimates taking the above factors into account have suggested up to 20–40 years of effective protection and compliance maybe needed to attain new steady states in a managed MPAs [13]. Finding long-term datasets to explore this has been difficult. Here, we take advantage of published data dating back to the establishment of the Saba Marine Park (SMP) in 1987 [22]–[24] to document the long-term response of the coral reef community to the spatial management (i.e. zones open and closed to fishing) of this relatively isolated Caribbean island.

Understanding the critical role that certain reef fishes play in ecosystem function and how they respond to changing habitat quality can provide key insights into the causes and consequences of long-term change in coral reef communities [18], [25]–[27]. For instance, the composition and presence of herbivorous fishes and their attendant bioeroding and grazing activities has been linked to two important aspects of ecosystem health: live coral recruitment and balanced coral-algal competition [25], [28], [29]. Functional linkages have also pointed to the habitat characteristics that underpin the presence of different components of coral reef fish diversity. For instance, a recent study found that structurally complex hard corals are the preferred shelter for large carnivorous fishes [30], which complements other studies finding that live coral and structural complexity is critical for the health and abundance of adult reef fishes [31]. Moreover, live hard coral has been directly linked to the survivorship and replenishment of juvenile reef fish [32]. Consequently, tracking changes in the abundance of fish trophic guilds and their required reef habitat can provide key indicators of ecosystem-relevant change.

In this study we examined the structure of a coral reef community following 21 years since the establishment of an isolated Caribbean MPA on Saba Island, Netherland Antilles. Combining published data with new recordings via the same methodology in a meta-analysis, our aims were threefold: (1) examine whether spatial protection has had an overall effect on the coral reef community since inception of the SMP, (2) explore temporal consistency in the affects of zoning (if any) on various components of the SMP coral reef community, and (3) examine the present distribution and abundance of herbivorous and carnivorous fish families and species across zones open and closed to fishing. In our discussion, we explore both the benefits and limitations of MPA-based local management of coral reefs in this region, and highlight the importance of effective monitoring for identifying and acting on coral reef vulnerability.

## Methods

**Figure 1:**
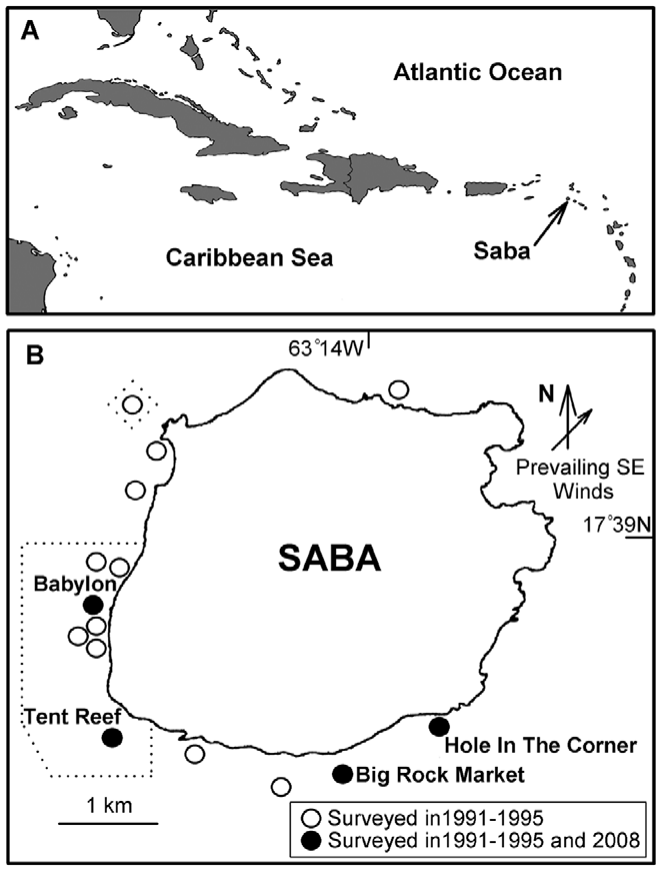
Map of Saba. (A) Location of Saba, Netherlands Antilles in the Caribbean Sea. (B) Study sites within the Saba Marine Park, Saba. All circles indicate study sites for the 1991–1995 censuses, with closed circles sites being those resurveyed in 2008. Dotted enclosure indicates the no-take zone “closed” to fishing.

**Figure 2:**
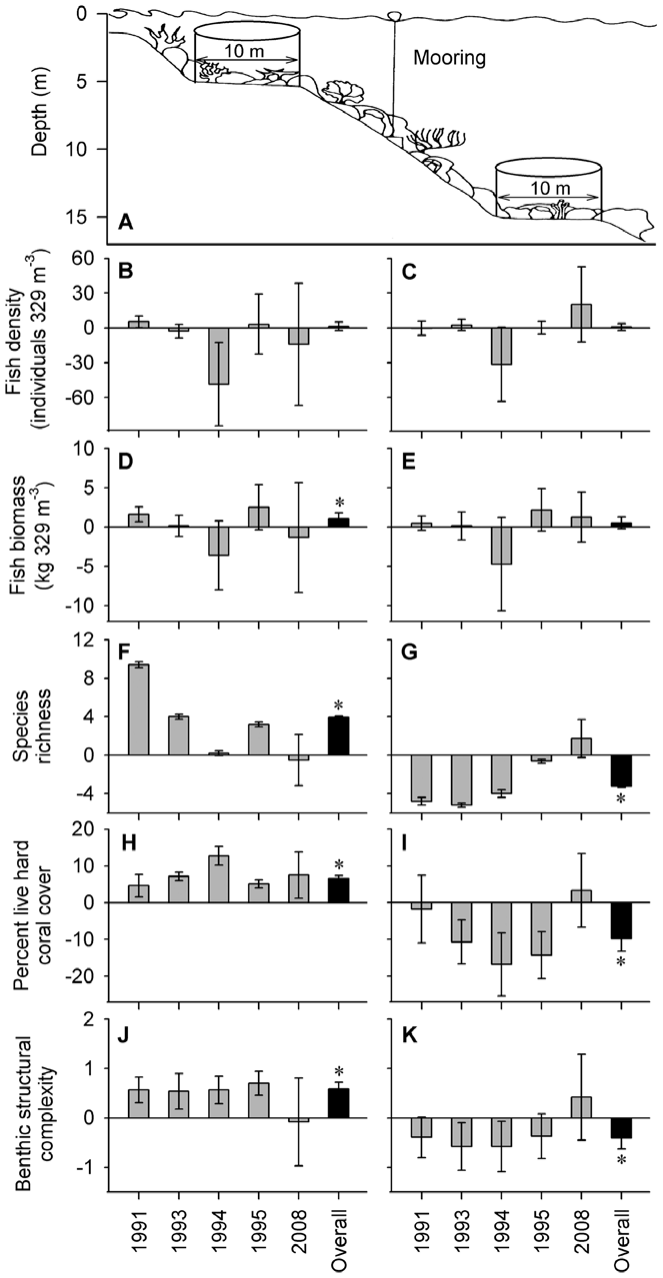
Mean difference in fish density, biomass, species richness and habitat structure among zones of the Saba Marine Park. (A) Schematic of point-census surveys conducted in shallow (5 m) and deep (15 m) habitats within a 329 m^3^ cylinder (10 m diameter at base). Mean difference (closed: open zones, ±95% confidence limits) for each of the five surveys (grey bars) and across all years (“overall”, black bar) in both shallow and deep habitats (left and right columns, respectively) for (B, C) fish density, (D, E) fish biomass, (F, G) fish species richness, (H, I) percent live hard coral cover and (J, K) benthic structural complexity index. Asterisks indicate a significant overall effect (Table 1). Data for years prior to 2008 sourced from Polunin and Roberts [22], Roberts [23] and Roberts and Hawkins [24].

Underwater visual censuses (UVCs) of fish abundance and habitat variables were conducted within the Saba Marine Park (SMP) surrounding Saba island (17°39′N, 63°14′W), Netherland Antilles (Fig. 1) using non-manipulative techniques that did not in any way harm the animals under observation, following protocols approved by The Executive Council of the Island Territory Saba (permit no. 0004/2010) for this specific project. The SMP covers a total area of 13 km^2^, with a no-take area of 4.29 km^2^ (approximately 33% of the SMP) [33]. The active volcano on Saba has some continuing geothermal activity, with steep sides to the island creating a narrow reef shelf that quickly descends to 60+ meters depth [34]. Contemporary surveys conducted during September - October 2008 complemented previously published data by Roberts and coauthors [22]–[24] by using identical methodology and study sites. Beginning shortly after the SMP establishment in 1987, the combined dataset encompasses the density and biomass of commercially targeted, reef-associated fishes (divided into families), as well as habitat variables measured in 1991, 1993, 1994, 1995 [22]–[24] and 2008 (present study). Original study sites were located using named moorings maintained by the Saba Conservation Foundation (SCF; Figures 1 and 2a). Working within tight logistical constraints, we chose a smaller subset of two sites within each of the closed (Tent Reef, Babylon) and open (Big Rock Market, Hole In The Corner) fishing zones in the SMP to minimize overlap between closely spaced sites, while maintaining similar environmental conditions such as wave exposure around this small island. Sites were also chosen for their proximity to the only harbor (Fort Bay) on Saba, where they are subject to repeated visits by the local diving and fisherpeople.

UVCs were made with the stationary point-count method (developed by Bonsack and Banerot [35] and utilized by Polunin and Roberts [22]; Roberts [23]; Roberts and Hawkins [24]) conducted by two observers on SCUBA within the crest (5 meter depth) and base (15 meter) habitats at each site (Fig. 2a). Each replicate survey position was selected at random once the target depth was reached. Each point-census involved placing a 10 m transect on the substratum and recording the species and total length (TL, estimated to nearest centimeter using a PVC fish measuring fork) of fish that were in, or passed through, a 10 meter wide by 5 meter high virtual cylinder over a 15-minute period. This was followed by a crawl census within the 10 meter footprint of the survey cylinder to locate and record small, benthic fishes. Care was taken to avoid recounting territorial individuals that remained within the cylinder throughout the sampling interval. Once each replicate fish survey was complete, the total percent cover of six different substratum types (live hard corals, dead coral, algae, gorgonians, sponges, and sand) were visually estimated within the 10 meter basal diameter of the survey cylinder. Degree of substratum structural complexity was also recorded using the point scale of previous studies [22]–[24], which ranged from 0–5: 0 = bare substratum, 1 = low and sparse relief, 2 = low but widespread relief, 3 = moderate complexity, 4 = high complexity with cave systems, 5 = extreme complexity with numerous caves and overhangs. This entire UVC procedure was repeated 6 times at each depth and site, with care taken to avoid spatial overlap among each replicate UVC. To minimize the impact of bias among past and present surveyors we consulted extensively with one of the previous observers (Prof. Callum Roberts) and took great care to follow their protocol exactly. Notably, the point count method employed has been found to be robust against many of the possible observer biases occurring in other survey techniques, such as belt transects, where variations in observer swimming speed, belt width estimation, and distance from the substratum underpin the majority of observer-based differences in fish censuses [35], [36]. However, previous examinations of observer-based differences in point census results have indicated an average 37% difference in fish counts among divers [22]. As such, any changes in fish densities and biomass between the old (1990s) and new (2008) censuses that were less than this range were treated with caution in our interpretation of the results. For the purposes of comparison with previously published data for the 1990s, surveys of fish in 2008 were pooled either: across all individuals (to calculate a total density and biomass for overall comparisons), or across all individuals within each family (for family-level comparisons), for each replicate UVC. Biomass was calculated by estimating the body mass of each individual fish counted during the surveys using the length-weight relationship equation W = aL^b^ described by Bonsack and Harper [37]. Constants (a, b) for the length-weight relationships for each species were sourced from FishBase [38]. Values for the mean and standard deviation of fish density and biomass (both overall and family-level), species richness, live hard coral cover and benthic structural complexity for each depth and zone during 1991–1995 were derived directly from reported figures [22]–[24] and combined with 2008 values to conduct a fixed-factor meta-analysis of the mean difference (MD) among closed and open zones of the SMP across the five survey periods (1991, 1993, 1994, 1995, 2008) for each component of the reef community. Tests for a significant overall effect of zoning (Z) on each reef component was calculated after weighting the MD for each survey by the precision (sample size and variance) in order to account for disparate sampling effort among zones and surveys (18–23 replicates per zone in 1991–95, 12 per zone in 2008) following Higgins and Green [39]. Significance levels were adjusted for multiple comparisons using the Bonferroni correction (k = 5 surveys). Graphical presentations of MD across surveys (±95% confidence limits) were used in combination with calculations of statistical heterogeneity (I^2^) following Higgins and Thompson [40] to explore temporal stability in the effects of zoning over time. Strong heterogeneity (indicated by high I^2^ and large **χ** ^2^) suggests that mean differences varied more across years than expected by random error alone (i.e. there was significant change in the magnitude and/or direction of the mean difference among zones from survey to survey). Further graphical examination of temporal trends in the density and biomass of “carnivorous” and “herbivorous” fishes (by merging family-level data according to the prior classification of Roberts [23], [24]) across closed and open zones were made for the shallow habitats (5 meters, where most significant overall effects were detected), alongside trends in species richness, mean percent cover of live hard coral and mean structural complexity index across all years. Finally, a contemporary analysis (using only 2008 data as species-level data unavailable for the 1990s) of whether differences exist in the biomass of the reef-associated fish species across depths and zones was conducted via three-way MANOVA, with zoning, site, and depth as fixed factors. Data were log_10_(x+1) transformed to minimize departures from normality and homoscedasticity. Statistical analyses and presentations were made with SPSS (version 19, IBM Corporation), RevMan (version 5.2, Cochrane Collaboration) and Sigmaplot (version 9, StatSoft Pty Ltd).

## Results

**Figure 3:**
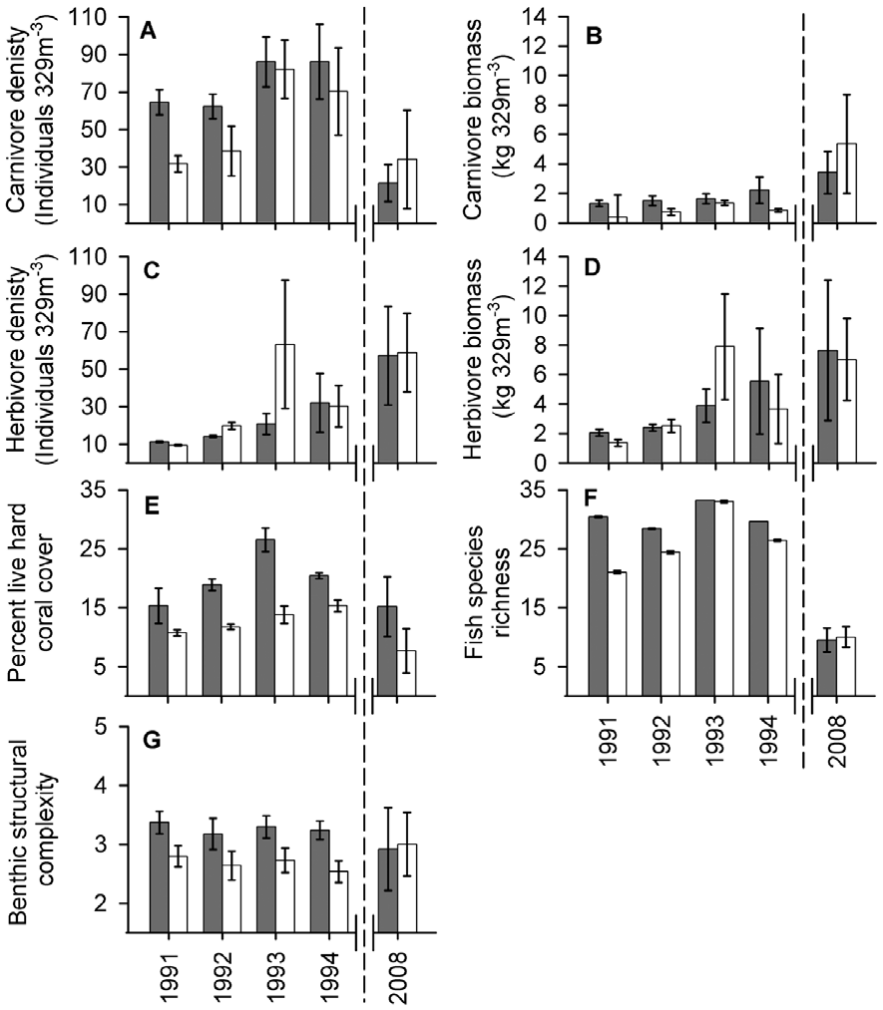
Temporal change in fish trophic guilds, species richness and habitat structure among zones of the Saba Marine Park. Comparison between closed (grey bars) and open (white bars) zones of the Saba Marine Park in terms of mean (±95% confidence limits) density and biomass (respectively) of (A, B) carnivorous and (C, D) herbivorous reef-associated fishes, alongside mean (E) percent cover of live hard coral, (F) species richness and (G) benthic structural complexity in shallow habitats (5 m depth) during 1991–1995 as compared to most recent (to right of dotted line) 2008 survey. Data for years prior to 2008 sourced from Polunin and Roberts [22], Roberts [23] and Roberts and Hawkins [24].

**Figure 4:**
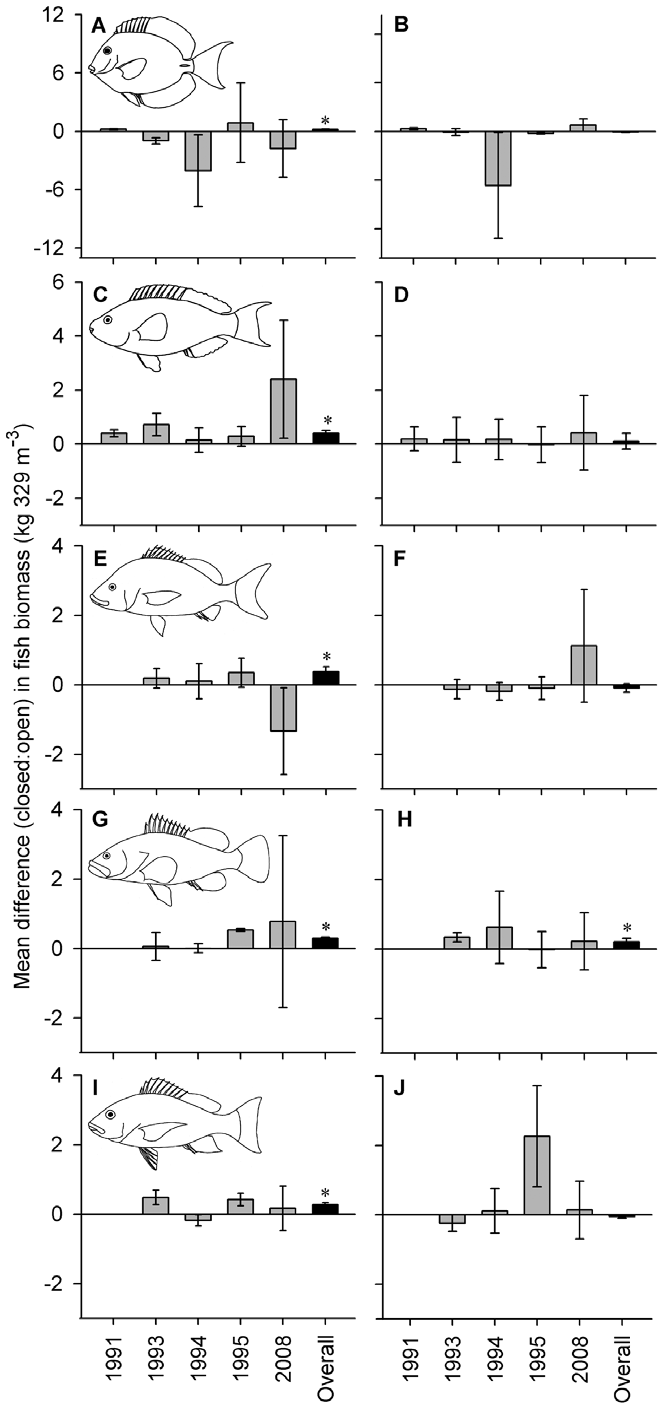
Mean difference in biomass of herbivorous and carnivorous fish families among zones of the Saba Marine Park. Mean difference (closed : open zones, ±95% confidence limits) is presented for each of the five surveys (grey bars) and across all years (“overall”, black bars) for both shallow (5 m) and deep (15 m) habitats (left and right columns, respectively) for the herbivorous fish families (A, B) Acanthuridae and (C, D) Scaridae, and the carnivorous fish families (E, F) Haemulidae, (G, H) Serranidae and (I, J) Lutjanidae. Asterisks indicate a significant overall effect (Table 2). Data for years prior to 2008 sourced from Polunin and Roberts [22], Roberts [23] and Roberts and Hawkins [24].

**Figure 5:**
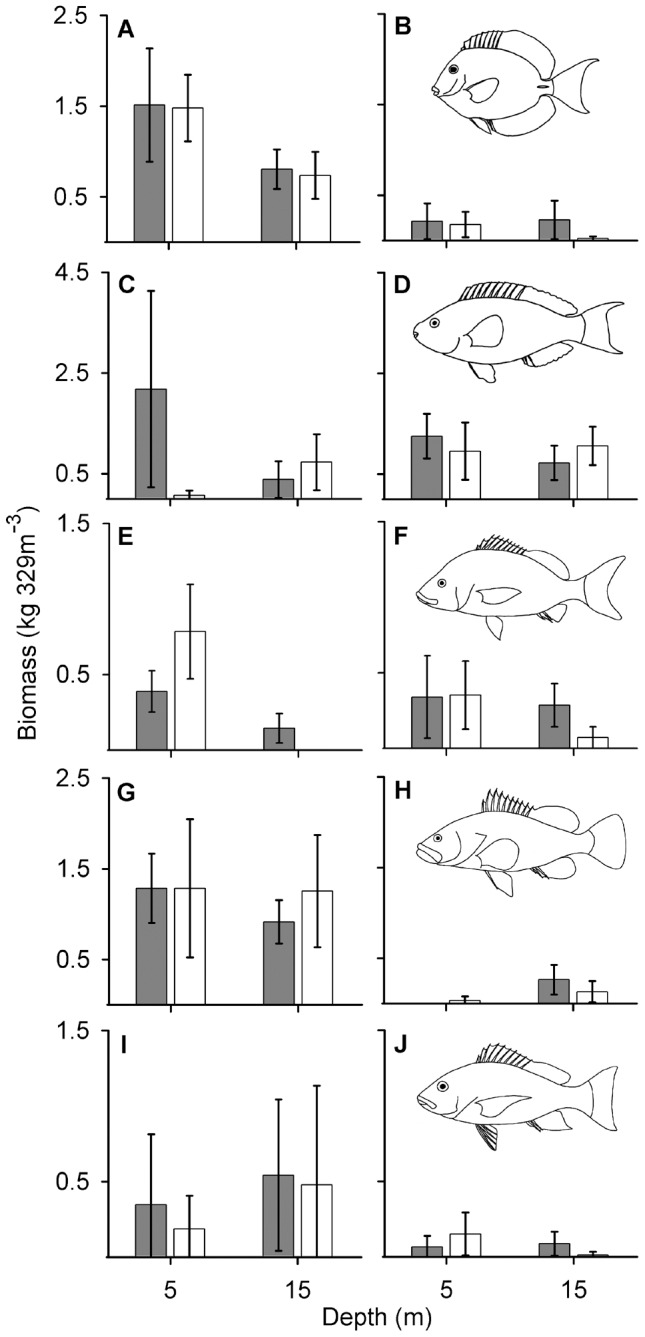
Biomass of reef-associated fish species among zones of the Saba Marine Park. Distribution of mean biomass (±95% confidence limits) of herbivorous (A–D) and carnivorous (E–J) fishes in 2008 across shallow (5 m) and deep (15 m) habitats in zones closed (grey bars) and open (white bars) to fishing. Species (functional role and trophic level indicated in parentheses [38]) are arranged in ascending trophic level: (A) *Acanthurus bahianus* (grazer, 2.0), (B) *Acanthurus chirurgus* (grazer, 2.0), (C) *Sparisoma viride* (excavator, 2.0), (D) *Sparisoma aurofrenatum* (scraper, 2.0), (E) *Haemulon carbonarium* (invertivore, 3.3), (F) *Haemulon flavolineatum* (invertivore, 3.3), (G) *Cephalopholis fulva* (carnivore, 4.1), (H) *Cephalopholis cruentata* (carnivore, 4.2.), (I) *Lutjanus apodus* (carnivore, 4.2) and (J) *Lutjanus mahogoni* (piscivore, 4.5).

Significant effects of spatial zoning within the Saba Marine Park were apparent for several aspects of the coral reef community, with strong heterogeneity (changes in the mean difference among zones across surveys) and temporal trends suggesting changes have occurred among survey years (Table 1, Fig. 2, Fig. 3). Across the five surveys we found a significant overall effect of zoning in shallow habitats with greater total fish biomass, species richness, percent live coral cover and benthic complexity (Fig. 2D, F, H and J), but no significant effect on fish density (Table 1). Significant overall effects in deeper habitats, however, indicated lower species richness, coral cover and benthic complexity in closed zones (Fig. 2G, I and K), with no significant effect on fish biomass or density (Table 1). Underlying these overall effects we found strong variability (heterogeneity) across survey years, particularly for fish density, biomass, species richness and live coral cover within shallow habitats (Table 1). Indeed, fish from the two trophic groups occurring within shallow-water habitats displayed divergent trajectories alongside changes in habitat structure (Fig. 3). Carnivorous fish displayed a 68% decline in density from 1995 to 2008 (Fig. 3A), while herbivorous fish density increased 49% over the same period (Fig. 3B), offset by only marginal increases in biomass (Fig. 3B and 3D, respectively). Concurrent to these trends, percent cover of live hard coral declined from up to 38% in 1994 to less than 10% in 2008 across all sites and zones (Fig.3E), while fish species richness was markedly lower in 2008 relative to the 1990s (Fig. 3F). Although marginally higher coral cover and herbivore density was apparent within zones closed to fishing, benthic structural complexity tended to converge towards a mean index of 3 across zones in 2008 (Fig. 3G). Family-level analyses revealed that significantly greater biomass in zones closed to fishing were apparent in all five fish families surveyed across all years, but mainly within shallow habitats (Table 2, Fig. 4). Serranids were the only family that displayed a significantly greater biomass in the deeper habitats (Table 2, Fig. 4H). Strong variability across surveys for two of these families in shallow habitats appear to be largely due to the significantly greater biomass of scarids and significantly less biomass of haemulids in closed zones during 2008 (Fig. 4C and Fig. 4E, respectively). Significant species-level variation in 2008 suggests inconsistent distributions of fish biomass across zones and depths with no apparent links to trophic level (Table 3, Fig. 5). This is supported by the fact that only some species displayed higher biomass in closed zones, and only at some depths, such as the herbivorous species *Acanthurus chirurgus* (15 m, Fig. 5B) and *Sparisoma viride* (5 m, Fig. 5C), and the carnivorous species *Haemulon carbonarium* (15 m, Fig. 5E) and *H. flavolineatum* (15 m, Fig. 5F). By contrast, there were no marked differences among zones for many other species, including the invertivore *Haemulon flavolineatum* (Fig. 5F), carnivore *Cephalopholis fulva* (Fig. 5G) and piscivore *Lutjanus mahogoni* (Fig. 5J).

**Table 1:** Overall effects and heterogeneity in the mean difference of reef-associated fishes and habitat structure among zones of the Saba Marine Park.

Variable	Overall effect (Z)		Heterogeneity (I^2^, χ^2^)	
	5 m	15 m	5 m	15 m
Fish density	0.68*p* = 0.50	0.49*p* = 0.62	67%, 12.05***p*** ** = 0.02**	33%, 5.94*p* = 0.20
Fish biomass	2.83***p*** **<0.001**	1.36*p* = 0.17	55%, 8.95***p*** ** = 0.06**	17%, 4.79*p* = 0.31
Fish species richness	56.65***p*** **<0.001**	46.89***p*** **<0.001**	100%, 2034.1***p*** **<0.001**	100%, 987.9***p*** **<0.001**
Live hard coral cover	17.84***p*** **<0.001**	14.04***p*** **<0.001**	88%, 32.82***p*** **<0.001**	72%, 14.04***p*** **<0.001**
Benthic structural complexity	8.51***p*** **<0.001**	3.61***p*** **<0.001**	0%, 3.16*p* = 0.53	10%, 4.43*p* = 0.35

Spanning five surveys (1991, 1993, 1994, 1995, 2008) at two depths (5 and 15 meters) among zones closed and open to fishing, the significant *p*-values for overall effect of zoning (Z, df = 4, α = 0.0125, after Bonferroni correction for multiple comparisons, k = 5) and variability across survey years (**χ** ^2^, df = 4, α = 0.10, [40]) are indicated in bold. Underlying data for 1991–95 sourced from Polunin and Roberts [22], Roberts [23] and Roberts and Hawkins [24].

**Table 2:** Summary of overall effects and temporal heterogeneity in the mean difference of fish biomass among zones of the Saba Marine Park.

Fish family	Overall effect (Z)		Heterogeneity (I^2^, χ^2^)	
	5 m	15 m	5 m	15 m
Herbivorous				
Acanthuridae	6.27***p*** **<0.001**	2.05*p* = 0.04	16%, 4.76*p* = 0.31	96%, 91.45***p*** **<0.001**
Scaridae	6.84***p*** **<0.001**	0.73*p* = 0.46	94%, 63.71***p*** **<0.001**	0%, 0.89*p* = 0.93
Carnivorous				
Haemulidae	5.50***p*** **<0.001**	1.45*p* = 0.15	90%, 40.08***p*** **<0.001**	0%, 3.24*p* = 0.52
Lutjanidae	9.22***p*** **<0.001**	1.64*p* = 0.10	44%, 7.18*p* = 0.13	37%, 6.30*p* = 0.18
Serranidae	16.79***p*** **<0.001**	4.07***p*** **<0.001**	99%, 318.23***p*** **<0.001**	64%, 10.98***p*** ** = 0.03**

Spanning five surveys (1991, 1993, 1994, 1995, 2008) of herbivorous and carnivorous fish families at depths (5 and 15 meters) among zones closed and open to fishing, the significant *p*-values for overall effect of zoning (Z, df = 4, α = 0.0125, after Bonferroni correction for multiple comparisons, k = 5) and variability across survey years (**χ** ^2^, df = 4, α = 0.05, [40]) are indicated in bold. Underlying data for 1991–95 sourced from Polunin and Roberts [22], Roberts [23] and Roberts and Hawkins [24].

**Table 3:** Summary of three-way MANOVA comparing biomass of reef-associated fishes across the Saba Marine Park.

Factor	Pillai’s trace	*F*	*p*-value
Zone	0.863	3.64	**<0.01**
Depth	0.878	4.16	**<0.01**
Site	0.801	2.32	0.06
Zone*Depth	0.642	1.03	0.49
Zone*Site	0.872	3.93	**<0.01**
Depth*Site	0.789	2.16	0.06
Zone*Depth*Site	0.700	1.35	0.28

Zone (closed and open to fishing), depth (5 m and 15 m) and site (two per zone) were fixed factors in a fully orthogonal design comprising a total of 48 point-count censuses of 27 fish species in 2008. Significant *p*-values (df = 26, 15, α = 0.05) are indicated in bold.

## Discussion

Despite effective spatial protection of the Saba Marine Park (SMP) by local managers for 21 years, we found no significant difference in overall fish density between open (fished) and closed (no-take) zones. While we did find marginally higher biomass of certain fish species in zones closed to fishing, this was generally restricted to shallow habitats and was tempered by an apparent decline in live hard coral cover between 1991–1995 and 2008. Notably, we found historically low carnivorous fish density across all zones in 2008, which was offset by marginal increases in their biomass, and slight increases in the density and biomass of herbivorous fishes. Such shifts in the habitat composition and trophic structure of the coral reef communities around Saba are cause for concern, as even subtle changes in community composition may have significant ecological consequences, and may indicate altered ecosystem resistance and resilience [41]. Based on lessons learned from coral reef collapses in the Caribbean and elsewhere [18], [20], [26], and increasing reports of region-wide trends of declining coral cover and related ecosystem-level consequences, the Saba reef ecosystem may be vulnerable to a regime shift to a less desirable community state. Although non-compliance and overfishing may be driving these changes around Saba [1], [26], [42], [43], shifting trends in fishing effort across the region, coupled with external stressors and habitat-loss suggest the observed community changes may be symptomatic of wider trends occurring throughout the Caribbean that are beyond the purview of local Marine Protected Area (MPA) management.

Evaluating whether spatial management of coral reefs is effective in meeting the intended goals of an MPA must be done in the context of time since establishment and zoning compliance. Overfishing has been a key explanation for regional declines in carnivorous fishes on Caribbean coral reefs with and without MPAs [18], [42]–[44]. Around Saba, regional shifts in fishing pressure have occurred since establishment of the Exclusive Economic Zone in 1996, with commercial catches declining sharply (93%) from 1987 to 2006 [33]. Coupled with relatively few recreational fishers, who are allowed to line fish from the shore and use baited traps or line-based trolling within the open zones (spearfishing is illegal everywhere), current fishing pressure around Saba appears to be light [45]. However, our findings of mixed effects of the SMP zoning on different aspects of the fish community, including little or no mean difference in fish density across zones and higher biomass only in shallow habitats, suggests we should consider the potential for non-compliance within the SMP. While poaching can cause rapid and lasting reductions of fish abundance in no-take areas [46], [47], daily patrols of SMP closed fishing zones by local rangers and frequent visits by local diving operations would suggest illegal fishing in closed zones close to the Saba harbour is unlikely. Moreover, the Saba Conservation Foundation (SCF) works closely with the small community of SMP users and has developed good rapport to foster voluntary compliance. Alternatively, the SMP no-take zones may simply be too small to fully contain routine movements of some top-level piscivores (haemulids, lutjanids and serranids), who could become exposed to fishing pressure in the adjacent open (fished) zones [48]. However, given that similar declines in carnivorous fish have been documented across the Caribbean [42], [43], this may be a regional phenomenon. Indeed, a recent meta-analysis found such fish declines were not well correlated to overfishing, but may arise from a time-lagged response to the loss of preferred coral reef habitats [49].

Shifts in the availability of live coral habitat could be a major driver of change in the reef-associated fish communities of Saba. Historical losses of live hard coral cover, such as the 38% decrease from 1995 to 2008 recorded for Saba, have been documented for coral reefs throughout the world and have often been attributed to factors such as thermal bleaching, hurricanes, pests and disease (e.g. [18], [49]–[54]). Saba and many places throughout the Caribbean have experienced increased hurricane activity over the past decade, with eight hurricanes passing with 100 km of the SMP between 1995–2008, versus only two hurricanes within the preceding 34 years [55]. As hurricanes often cause substantial hard coral loss (e.g. 18, 34, 56), the maintenance of live coral on a reef is dependent upon the health of adults and processes of replenishment. Such recovery has often been compromised by other stressors, like coral disease and thermal bleaching [57], [58], with up to 80% of the reefs around Saba experiencing mass bleaching as part of the Caribbean-wide events in 1998 and 2005 [34], [53], [59]. Such coral loss can substantially alter fish community structure and species richness on coral reefs, with numerous studies documenting significant declines in the abundance of adult reef fishes who depend on live coral for food and/or habitat when reefs incur major losses of live coral cover [31], [32], [60]. Fish population replenishment can also be jeopardised by losses of live coral, since many coral reef fishes settle preferentially into live hard corals and will not colonise reef patches without live coral [32], [61]. Notably, we did find significantly greater fish biomass and species richness in the shallow habitats closed to fishing, which also tended to have higher percent cover of live coral relative to open zones. Conversely, deep habitats within the no-take zone tended to have lower coral cover and lower species richness relative to deep habitats open to fishing. While we lack historical information on the species-specific changes that have occurred within the SMP over time, further work on the current patterns of habitat-specificity of different fish species on the Saba reefs may shed light on how habitat-driven mechanisms are shaping zoning affects within the SMP. In the interim, we suggest SMP managers may consider rehabilitation of live hard coral cover within deep habitats closed to fishing in order to rebalance the presence of live coral habitat across closed and open zones. Monitoring how the reef-associated fishes respond to such management intervention could yield important insights into habitat-driven influences on MPA effectiveness.

Losses in live coral alongside changes in fish community structure can have serious consequences for the health of coral reefs and their capacity to resist and rebound from disturbance events. Around Saba, we found 68% less carnivorous fish across all sites and zones of the SMP in 2008 relative to the 1990s, offset by only marginal increases in their biomass, and slight increases in herbivorous fish across the same period. There was no clear evidence that zoning has played a role in these trophic-level trends, as we found that all families tended to display greater biomass in closed zones across all survey years, with relatively light variability across survey years. The notable exceptions, however, was that in 2008 a substantially greater biomass of herbivorous scarids were recorded in closed zones, while there was markedly lower biomass of carnivorous haemulids. Such dynamic shifts in community trophic structure can produce wider ecosystem effects through trophic cascades, such as the urchin overgrazing of the benthos on reefs bereft of predatory fishes (e.g. [18]), which erodes the capacity for these reefs to obtain new coral recruits. While reefs of the SMP may be in a vulnerable state that is susceptible to a regime shift, like many other coral reefs in the Caribbean [18], [26], [62], this will depend on the presence and diversity of a range of key functional groups, such as grazers, scrapers and excavators, that play a critical role in balancing coral-seaweed competition and facilitate coral recruitment [18], [26], [62]. Caribbean reefs like Saba can be particularly prone to community regime shifts, due to low diversity among and within key functional groups [26], [62], [63]. Indeed, Saba has just one abundant species of excavating fish, *Sparisoma viride*, which plays a pivotal role in the bioerosion and sculpturing of reefs to facilitate the removal of dead coral skeleton and prime the reef for new coral recruits [29], [64], [65]. Given decades of decline in live coral cover, it would seem this functional role is critically important to the maintenance of SMP coral reefs as well as others throughout the Caribbean [62]. Similarly low diversity, and therefore limited functional redundancy, also exists within the group of fishes that graze and scrape algae from reef surfaces around Saba [62], [63], although grazing by other herbivorous members of the Saba coral reef community (e.g. urchins) remains a large unknown and needs to be explored further [18], [29]. Given the multitude of possible regime shift drivers that are operating around Saba (e.g. hurricanes, coral disease, bleaching), it is imperative that management focus their strategies towards the protection of the few critical species, such as *Sparisoma viride*, to bolster reef resistance to regime shifts in the face of large-scale disturbances.

Long-term change within the coral reef communities of Saba and other parts of the Caribbean highlight the effective scope and limitations of local-scale spatial management, and point to the need for targeted strategies that bolster coral reefs against large-scale threats [4]. Recognising that MPAs alone cannot prevent declines in coral cover arising from thermal bleaching and other disturbances arising from global climate change [4], [32], [57], managers must implement strategies that maintain key functional groups and remediate critical habitats to assist reefs to be resilient [4], [27], [66], [67]. Our evidence suggests that spatial management can produce positive effects, but also provides a warning that Saba reefs are indicative of those throughout the Caribbean in being in a vulnerable state, with declining live coral and shifting fish trophic structure [4], [27], [26]. If changes in harvesting pressure were to target a critical functional group (i.e. herbivores and bioeroders), we could see a regime shift of these reefs to a less desirable community state. By providing targeted local protection to critical components of the fish fauna, plus key interventions to stabilise and improve live coral habitat, managers could help protect reefs against disturbances and assist their subsequent recovery [26], [62], [66], [67]. Using this resilience-based approach, we can complement current spatial management of coral reef ecosystems to reinforce natural feedbacks that promote resistance and resilience to the large-scale stressors affecting the region [27].

## References

[1] Russ GR (2002) Yet another review of marine reserve as reef fishery management tools In: Sale PF editor. Coral reef fishes: dynamics and diversity in a complex. Academic Press, San Diego. 421–443.

[2] Gaines SD, White C, Carr MH, Palumbi SR (2010) Designing marine reserve networks for both conservation and fisheries management. Proc Natl Acad Sci 107: 18286–18293. 20200311 10.1073/pnas.0906473107 PMC2972919

[3] McCook LJ, Ayling T, Cappo M, Choat HJ, Evans RD, et al (2010) Adaptive management of the Great Barrier Reef: a globally significant demonstration of the benefits of networks of marine reserves. Proc Natl Acad Sci 107: 18278–18285. 20176947 10.1073/pnas.0909335107 PMC2972947

[4] Graham NAJ, Ainsworth TD, Baird AH, Ban NC, Bay LK, et al (2011) From microbes to people: tractable benefits of no-take areas for coral reefs. Oceanogr Mar Biol Annu Rev 49: 105–136.

[5] Halpern BS (2003) The impact of marine reserves: do reserves work and does reserve size matter? Ecol Appl 13: S117–S137.

[6] McClanahan TR, Graham NAJ, Calnan JM, MacNeil MA (2007) Toward pristine biomass: reef fish recovery in coral reef marine protected areas in Kenya. Ecol Appl 17: 1055–1067. 17555218 10.1890/06-1450

[7] Claudet J, Osenberg CW, Benedetti-Cecchi L, Domenici P, García-Charton JA, et al (2008) Marine reserves: size and age matter. Ecol Lett 11: 481–489. 18294212 10.1111/j.1461-0248.2008.01166.x

[8] Lester SE, Halpern BS, Grorud-Colvert K, Lubchenco J, Ruttenberg BI, et al (2009) Biological effects within no-take marine reserves: a global synthesis. Mar Ecol Prog Ser 384: 33–46.

[9] Pollnac R, Christie P, Cinner JE, Dalton T, Daw TM, et al (2010) Marine reserves as linked social–ecological systems. Proc Natl Acad Sci 107: 18262–18265. 20176948 10.1073/pnas.0908266107 PMC2972944

[10] Selig ER, Bruno JF (2010) A global analysis of the effectiveness of marine protected areas in preventing coral loss. PLoS ONE 5: e9278. 20174644 10.1371/journal.pone.0009278 PMC2822846

[11] Molloy PP, McLean IB, Côte IM (2009) Effects of marine reserve age on fish populations: a global meta-analysis. J Appl Ecol 46: 743–751.

[12] Babcock RC, Shears NT, Alcala AC, Barrett NS, Edgar GJ, et al (2010) Decadal trends in marine reserves revel differential rates of change in direct and indirect effects. Proc Natl Acad Sci 107: 18256–18261. 20176941 10.1073/pnas.0908012107 PMC2972978

[13] Russ GR, Alcala AC (2010) Decadal-scale rebuilding of predator biomass in Philippine marine reserve. Oecologia 163: 1103–1106. 20593200 10.1007/s00442-010-1692-3

[14] Williamson DH, Russ GR, Ayling AM (2004) No-take marine reserves increase abundance and biomass of reef fish on inshore fringing reefs of the Great Barrier Reef. Environ Conserv 31: 149–159.

[15] Almany GR, Connolly SR, Heath DD, Hogan JD, Jones GP, et al (2009) Connectivity, biodiversity conservation and the design of marine reserve networks for coral reefs. Coral Reefs 28: 339–351.

[16] Berumen ML, Almany GR, Planes S, Jones GP, Saenz-Agudelo P, et al (2012) Persistence of self-recruitment and patterns of larval connectivity in a marine protected area network. Ecol Evol 2: 444–452. 22423335 10.1002/ece3.208 PMC3298954

[17] Harrison HB, Williamson DH, Evans RD, Almany GR, Thorrold SR, et al (2012) Larval export from marine reserves and the recruitment benefit for fish and fisheries. Curr Biol 22: 1023–1028. 22633811 10.1016/j.cub.2012.04.008

[18] Hughes TP (1994) Catastrophes, phase shifts, and large-scale degradation of a Caribbean coral reef. Science 265: 1547–1551. 17801530 10.1126/science.265.5178.1547

[19] Connell JH, Hughes TP, Wallace CC (1997) A 30-year study of coral abundance, recruitment, and disturbance at several scales in space and time. Ecol Monogr 67: 461–488.

[20] Graham NAJ, Wilson SK, Jennings S, Polunin NVC, Robinson J, et al (2007) Lag effects in the impacts of mass coral bleaching on coral reef fish, fisheries, and ecosystems. Conserv Biol 21: 1291–1300. 17883494 10.1111/j.1523-1739.2007.00754.x

[21] Alvarez-Filip L, Dulvy NK, Gill JA, Côté IM, Watkinson AR (2009) Flattening of Caribbean coral reefs: region-wide declines in architectural complexity. Proc Roy Soc B 276: 3019–3025. 10.1098/rspb.2009.0339 PMC2817220 19515663

[22] Polunin NVC, Roberts CM (1993) Greater biomass and value of target coral-reef fishes in two small Caribbean marine reserves. Mar Ecol Prog Ser 100: 167–176.

[23] Roberts CM (1995) Rapid build-up of fish biomass in a Caribbean marine reserve. Conserv Biol 9: 815–826.

[24] Roberts CM, Hawkins JP (1995) Status of reef fish and coral communities of the Saba Marine Park –1995. ECC Tech Rep. Eastern Caribbean Center, University of the Virgin Islands, St. Thomas, Virgin Islands USA.

[25] Bellwood DR, Hoey AS, Choat JH (2003) Limited functional redundancy in high diversity systems: resilience and ecosystem function on coral reefs. Ecol Lett 6: 281–285.

[26] Bellwood DR, Hughes TP, Folke C, Nyström M (2004) Confronting the coral reef crisis. Nature 429: 827–833. 15215854 10.1038/nature02691

[27] Hughes TP, Graham NAJ, Jackson JBC, Mumby PJ, Steneck RS (2010) Rising to the challenge of sustaining coral reef resilience. Trends Ecol Evol 25: 633–642. 20800316 10.1016/j.tree.2010.07.011

[28] Ledlie MH, Graham NAJ, Bythell JC, Wilson SK, Jennings S, et al (2007) Phase shifts and the role of herbivory in the resilience of coral reefs. Coral Reefs 26: 641–653.

[29] Sandin SA, McNamara DE (2012) Spatial dynamics of benthic competition on coral reefs. Oecologia 168: 1079–1090. 22009340 10.1007/s00442-011-2156-0

[30] Kerry JT, Bellwood DR (2012) The effect of coral morphology on shelter selection by coral reef fishes. Coral Reefs 31: 415–424.

[31] Wilson SK, Polunin NVC, Pratchett MS, Jones GP, Polunin NVC (2006) Multiple disturbances and the global degradation of coral reefs: are reef fishes at risk or resilient? Glob Change Biol 12: 2220–2234.

[32] Jones GP, McCormick MI, Srinivasan M, Eagle JV (2004) Coral decline threatens fish biodiversity in marine reserves. Proc Natl Acad Sci 101: 8251–8253. 15150414 10.1073/pnas.0401277101 PMC419589

[33] Sea Around Us Project (2012) Global catch database. Pew Environment Group, Fisheries Centre, Aquatic Ecosystems Research Laboratory, University of British Columbia, Vancouver, B. C. Canada. Available: www.seaaroundus.org. Accessed 2012 May 7.

[34] Wilkinson C (2008) Status of the coral reefs of the world: 2008. Global Coral Reef Monitoring Network, and Reef and Rainforest Research Centre, Townsville, Australia.

[35] Bonsack JA and Bannerot SP (1986) A Stationary Visual Census Technique for Quantitatively Assessing Community Structure of Coral Reef Fishes. NOAA Tech Rep: NMFS 41. NOAA, National Marine Fisheries Service, Miami Florida.

[36] Cheal AJ, Thompson AA (1997) Comparing visual counts of coral reef fish: implications of transect width and species selection. Mar Ecol Prog Ser 158: 241–248.

[37] Bonsack JA, Harper DE (1988) Length-weight relationships of selected marine reef fishes from the Southeastern United States and the Caribbean. NOAA Tech Memo NMFS-SEFC: 215. NOAA, National Marine Fisheries Services, South East Fisheries Center, Miami Florida.

[38] Froese R, Pauly D (2007) FishBase. World Wide Web electronic publication. Available: www.fishbase.org. Accessed 2012 April 4.

[39] Higgins JPT, Green S (2009) Cochrane Handbook for Systematic Reviews of Interventions (version 5.0.2). The Cochrane Collaboration. Available: www.cochrane-handbook.org.

[40] Higgins JPT, Thompson SG (2002) Quantifying heterogeneity in a meta-analysis. Stat Med 21: 1539–1558. 12111919 10.1002/sim.1186

[41] Berumen ML, Pratchett MS (2006) Recovery without resilience: persistent disturbance and long-term shifts in the structure of fish and coral communities at Tiahura Reef, Moorea. Coral Reefs 25: 647–653.

[42] Jackson JBC, Kirby MX, Berger WH, Bjorndal KA, Botsford LW, et al (2001) Historical overfishing and the recent collapse of coastal ecosystems. Science 293: 629–638. 11474098 10.1126/science.1059199

[43] Stallings CD (2009) Fishery-independent data reveal negative effect of human population density on Caribbean predatory fish communities. PLoS One 4: e5333. 19421312 10.1371/journal.pone.0005333 PMC2672166

[44] Westera M, Lavery P, Hyndes G (2003) Differences in recreationally targeted fishes between protected and fished areas of a coral reef marine park. J Exp Mar Biol Ecol 294: 145–168.

[45] Toller W, Debrot AO, Vermeij MJA, Hoetjes PC (2010) Reef fishes of Saba Bank, Netherlands Antilles: assemblage structure across a gradient of habitat types. PLoS One 5: e9207. 20502637 10.1371/journal.pone.0009207 PMC2873942

[46] Russ GR, Alcala AC (1998) Natural fishing experiments in marine reserves 1983–1993: roles of life history and fishing intensity in family responses. Coral Reefs 17: 399–416.

[47] Jupiter SD, Weeks R, Jenkins AP, Egli DP, Cakacaka A (2012) Effects of a single intensive harvest event on fish populations inside a customary marine closure. Coral Reefs 31: 321–334.

[48] Zeller DC, Russ GR (1998) Marine reserves: patterns of adult movement of the coral trout *Plectropomus leopardus* (Serranidae). Can J Fish Aquatic Sci 55: 917–924.

[49] Paddack MJ, Reynolds JD, Aguilar C, Appeldoorn RS, Beets J, et al (2009) Recent region-wide declines in Caribbean reef fish abundance. Curr Biol 19: 1–6. 19303296 10.1016/j.cub.2009.02.041

[50] Gardner TA, Côté IM, Gill JA, Grant A, Watkinson AR (2003) Long-term region-wide declines in Caribbean corals. Science 301: 958–960. 12869698 10.1126/science.1086050

[51] Côté IM, Gill JA, Gardner TA, Watkinson AR (2005) Measuring coral reef decline through meta-analyses Philos Trans R Soc B. 360: 385–395. 10.1098/rstb.2004.1591 PMC1569462 15814352

[52] Bruno J, Selig ER (2007) Regional decline of coral cover in the Indo-Pacific: timing, extent, and subregional comparisons. PLoS ONE 2: e711. 17684557 10.1371/journal.pone.0000711 PMC1933595

[53] Eakin CM, Morgan JA, Heron SF, Smith TB, Liu G, et al (2010) Caribbean corals in crisis: record thermal stress, bleaching, and mortality in 2005. PLoS ONE 5: e13969. 21125021 10.1371/journal.pone.0013969 PMC2981599

[54] De’ath G, Fabricius KE, Sweatman H, Puotinen M (2012) The 27-year decline of coral cover on the Great Barrier Reef and its causes. Proc Nat Acad Sci 107: 17995–17999. 10.1073/pnas.1208909109 PMC3497744 23027961

[55] van Dijken G (2012) Storm Carib: Caribbean Hurricane Network. Available: http://stormcarib.com/climatology/TNCS_dec_isl.htm. Accessed 2012 May 17.

[56] Chael AJ, Coleman G, Delean S, Miller I, Osborne K, et al (2002) Responses of coral and fish assemblages to a severe but short-lived tropical cyclone on the Great Barrier Reef, Australia. Coral Reefs 21: 131–142.

[57] Huntington BE, Karnauskas M, Lirman D (2011) Corals fail to recover at a Caribbean marine reserve despite ten years of reserve designation. Coral Reefs 30: 1077–1085.

[58] Prechet WF (2002) Endangered *acroporid* corals of the Caribbean. Coral Reefs 21: 41–42.

[59] Goreau T, McClanahan T, Hayes R, Strong AE (2000) Conservation of coral reefs after the 1998 global bleaching event. Conserv Biol 14: 5–15.

[60] Wilson SK, Burgess SC, Cheal AJ, Emslie M, Fisher R, et al (2008) Habitat utilization by coral reef fish: implications for specialists vs. generalists in a changing environment. J Anim Ecol 77: 220–228. 18081778 10.1111/j.1365-2656.2007.01341.x

[61] Coker DJ, Graham NAJ, Pratchett MS (2012) Interactive effects of live coral and structural complexity on the recruitment of reef fishes. Coral Reefs 31: 919–927.

[62] Mumby PJ (2009) Phase shifts and the stability of macroalgal communities on Caribbean coral reefs. Coral Reefs 28: 761–773.

[63] Bellwood DR, Hoey AS, Choat JH (2003) Limited functional redundancy in high diversity systems: resilience and ecosystem function on coral reefs. Ecol Lett 6: 281–285.

[64] Bruggemann JH, van Kessel AM, van Rooij JM, Breeman AM (1996) Bioerosion and sediment ingestion by the Caribbean parrotfish *Scarus vetula* and *Sparisoma viride*: implications of fish size, feeding mode and habitat use. Mar Ecol 134: 59–71.

[65] Cardoso SC, Soares MC, Oxenford HA, Côté IM (2006) Interspecific differences in foraging behavior and functional role of Caribbean parrotfish. Mar Biodivers Rec 2: 1–6.

[66] Nyström M, Graham NAJ, Lokrantz J, Norström AV (2008) Capturing the cornerstones of coral reef resilience: linking theory to practice. Coral Reefs 27: 795–809.

[67] Halford A, Caley MJ (2009) Towards an understanding of resilience in isolated coral reefs. Glob Change Biol 15: 3031–3045.

